# Fatigue and related variables in bladder cancer treatment – Longitudinal pilot study

**DOI:** 10.1016/j.heliyon.2024.e35995

**Published:** 2024-08-08

**Authors:** Agata Zdun – Ryżewska, Teresa Gawlik-Jakubczak, Agnieszka Trawicka, Paweł Trawicki

**Affiliations:** a Division of Quality of Life Research, Department of Psychology, Faculty of Health Sciences, Medical University of Gdansk, Poland; b Department of Urology, Faculty of Medicine, Medical University of Gdansk, Poland; c Department of Developmental Support Psychology, Faculty of Psychology, University of Social Sciences and Humanities, Sopot, Poland; d Division of Contemporary History and Political Thought, Faculty of Social Sciences, Institute of Political Sciences, University of Gdańsk, Poland

**Keywords:** Bladder cancer, Cancer-related fatigue, Cystectomy

## Abstract

Fatigue is a significant problem in patients with bladder cancer treated by radical cystectomy. This pilot study evaluated fatigue and related variables during a treatment period. Four measurements were made, the first 1 month after the cystectomy, and the next three at an interval of about 3 months each (at 4 months, 7 months, and 10 months after the surgery). In addition to the author's questionnaire (sociodemographic variables and a question about the impact of the disease on the patient's life), the FACIT-F Fatigue (to measure fatigue), NCCN/FACT FBISI-18, version 2 (symptoms, general condition of the patient), HADS (depression, anxiety, and irritability) measures were used. In this study, 21 patients participated in all four measurement periods. The fatigue intensity increased significantly between the first and second measurements and gradually decreased between the third and fourth measurements.

As the severity of fatigue increases, can be observed an increase in the sense of the impact of the disease on the patient's life in all except the first measurement.

The study revealed statistically significant correlations between fatigue and experiencing symptoms of cancer and treatment at each stage of the study, with the strongest correlations in the second and fourth measurements regarding symptoms of cancer and a stronger correlation in the second compared to the first measurement regarding side effects. At each stage of measurement, the experience of dizziness, lack of appetite, feeling of being sick, and feeling of annoyance from treatment side effects were statistically significantly correlated with fatigue. The intensity of fatigue correlated with the feeling of experiencing difficulties in meeting the needs of the family due to the physical condition in the first measurement (Rho = 0.76), a sense of weakness (Rho = 0.92) and sleepiness (Rho = 0.72) in the second measurement, pain in the third (Rho = 0.77). The greatest number of correlates of fatigue were described in the fourth measurement (all symptoms of cancer and side effects except losing weight).

Stress, anxiety, depression and irritability were correlated with fatigue at each of the stages of research except the first one (without differences between the correlation coefficients in the second, third and fourth measurements). Significantly lower levels of fatigue characterised patients who survived over 6 months after the end of the study compared to the first three measurements.

## 1 Introduction

Bladder cancer is a common malignancy, estimated to be among the 10 most frequently diagnosed cancers worldwide, with approximately 200,000 cases reported annually [1]. Despite its prevalence, there remains a need for further research in this area. Scientific publications predominantly focus on other cancers, such as breast, prostate and kidney cancer [2]. A similar disparity exists in cancer-related fatigue research, where breast cancer patients constitute the most studied group. Interestingly, bladder cancer patients exhibit the highest prevalence of emotional fatigue compared to 15 other cancer types [3].

Cancer-related fatigue is a pervasive and often overlooked symptom in cancer medicine. It manifests as a complex syndrome with distinct physical, mental, and emotional components, stemming from various etiologies. Patients themselves frequently describe it as chronic and abnormal tiredness, accompanied by a disproportionate decrease in performance that can not be easily alleviated by rest [4]. Notably, minimal fatigue serves as a positive cancer prognostic factor for time-to-progressive disease and for time-to-treatment in patients with advanced or metastatic bladder cancer [5]. Post-cystectomy patients, who undergo the most radical treatment method, report even greater fatigue, along with appetite loss, weight reduction, and a significant decline in role-functioning. Older male patients, in particular, experience sleep disturbances, emotional problems and challenges following surgery, affecting their overall quality of life [6].

## 2 Theory

The exploration of variables associated with fatigue and its potential causes occupies a significant portion of the oncology and psycho-oncology literature. Such research is crucial for designing specific methods to help cancer patients cope with fatigue.

In the scientific literature within the field of oncology, there exists a concept known as a symptom cluster, which refers to symptoms that are related in a specific way. Examples of such variables include the frequently studied relationship between fatigue and pain [7,8], as well as the connections between fatigue and other symptoms such as drowsiness, nausea, decreased appetite [9], and sleep [10].

Numerous studies conducted by other researchers highlighted the presence of chronic fatigue in cancer patients, often intertwined with both physical and psychological symptoms. For instance, one study demonstrated that fatigue in cancer patients with prostate cancer was influenced not only by urinary dysfunction and pain (physical symptoms) but also by depression (a mental health variable) [11].

Despite numerous studies conducted in this area, there remains a need to establish connections between fatigue and symptoms associated with the progression and treatment of cancer. Additionally, the mechanism explaining these associations requires further investigation [12]. A recently published systematic review examining the quality of life among bladder cancer patients across different treatment stages, emphasised the necessity for further research in the area of patients's functioning, in terms of quality of life and related variables [13].

The present pilot study aimed to investigate the intensity of fatigue experienced by patients diagnosed with bladder cancer who underwent cystectomy within approximately one year after surgery. Our goal was also to identify variables associated with fatigue, including both subjectively assessed health status and mental functioning. These findings might lay the groundwork for developing a psychoeducational program to help patients manage fatigue. To achieve these objectives, we implemented a rigorous longitudinal research design. Four measurement points were scheduled, during which each patient's fatigue level and potentially correlated variables were assessed. These variables were categorized into two groups: symptoms and complaints experienced by patients related to the disease and its treatment, and factors associated with functioning and mental well-being.

## 3 Material and methods

### 3.1 Procedure

This study received approval from a local bioethics committee without any ethical objections (Resolution No. NKBBN/452/2022). Patients from a university clinical centre urology clinic were invited to participate in the study. Participation was entirely voluntary, with the option to withdraw at any time. Informed consent was obtained from each participant.

The research followed a longitudinal design involving multiple assessments of the same variables to explore their intensity, severity, and interrelationships across different stages of treatment. The patients completed a total of four questionnaires. The first was administered within 1 month after surgery, followed by three additional measurements (approximately 3months apart). Data collection concluded in 2022.

The study, part of a large project, represents the first publication derived from a comprehensive collection of materials.

### 3.2 Methods

#### 3.2.1 Self-designed questionnaire

This study employed a self-designed questionnaire to collect socio-demographic data, including age, gender, employment, financial situation, marital status, and place of residence. An additional question in this section also covered the extent to which the disease affects everyday life, also on a scale of 0 (“not at all”), to 10 (“very much affects my life”).

#### 3.2.2 Functional assessment of chronic illness therapy – fatigue (FACIT-F fatigue, version 4.0)

Fatigue was assessed using a 13-item scale specifically designed for adult cancer patients [14]. Participants responded to each statement using a 5-point Likert scale. The questionnaire is brief and easy to administer, and its reliability and validity have been established [15,16]. Permission was obtained to use the translated scale for non-commercial scientific purposes.

#### 3.2.3 National comprehensive cancer network/functional assessment of cancer therapy bladder symptom index (NCCN/FACT FBISI-18, version 2)

The questionnaire was developed to assess the symptoms, overall condition, and well-being specifically for patients with bladder cancer. Participants respond to questions using a 5-point Likert scale ranging from “not at all” to “very much”. The psychometric properties of the questionnaire are satisfactory, enabling its use in clinical practice and research [17,18]. For this study, we employed the general score of the questionnaire and two subscale scores in detail regarding the symptoms of the disease in the type of cancer studied.

#### 3.2.4 The hospital anxiety and depression scale (HADS)

The HADS is a questionnaire designed to assess symptoms of depression (depressed mood, loss of enjoyment, sadness, loss of interests) and anxiety (tension, nervousness) in a group of patients [19]. It has been validated for screening these symptoms in patients with chronic, serious somatic diseases and exhibits excellent psychometric properties. The Polish version includes two additional questions about „annoyance” [20].

#### 3.2.5 Statistical analyses

Due to the small sample size, non-parametric methods were employed. The analyses were conducted using the statistical programme Statistica version 13.3 (TIBCO Software Inc). The Kruskal-Wallis test was used for comparing multiple groups (with Bonferroni correction for multiple comparisons), the Mann-Whitney *U* test for comparing two variables, and Spearman's correlation coefficient for describing relationships between variables. Recommendations for interpreting the strength of correlation coefficients were taken from the guidelines proposed by authors working in the field of medical sciences [21]. Correlation comparisons between measurements were also performed (between measurement moment comparisons). The threshold for statistical significance was set at p-value <0.05.

### 3.3 Participants

#### 3.3.1 Sociodemographic characteristics

Twenty-one participants took part in all four measurements. Socio-demographic characteristics are presented in Table 1. Mostly retired, with a fairly good assessment of their financial situation, the majority of the study group were elderly men who were in a relationship with another person. Educational attainment was nearly evenly disbursed across the study group.

**Table 1:** Socio-demographic characteristic of the study group (N = 21).

Variable	Mean, SD N(%)
Age	M 69.14
	SD7.41
Gender	
Male	14 (67 %)
Female	7 (33 %)
Marital status	
Marriage or partnership	14 (67 %)
Widowhood/divorce	7 (33 %)
Education	
Higher	6 (28.5 %)
Secondary	9 (43 %)
Vocational and primary	6 (28.5 %)
Employment	
Hired	3 (14 %)
Unemployed	1 (5 %)
Pension	2 (10 %)
Retired	15 (71 %)
Financial situation	
Very good	1 (5 %)
Good	13 (62 %)
Average	6 (28 %)
Bad	1 (5 %)
Number of TURT proceduresBefore radical surgery	M 2.09SD 1.6
Death data	8 (38.1 %)

#### 3.3.2 Clinical characteristics of the study group

Earlier diagnosed with bladder cancer by an oncologist in charge of the patient's treatment, 21 patients participated in this study. Patients in the earlier treatment period underwent a total of 43 TURT (transurethral resection of tumour) procedures, with an average of two procedures per patient (M 2.09, SD 1.6). All patients were qualified for radical removal of the bladder. In men, the procedure involves the removal of the bladder, prostate and seminal vesicles, and surrounding lymph nodes. In women, the bladder, uterus with appendages and vagina are removed, as well as the surrounding lymph nodes. In 20 of the patients, after the operation the bladder cancer was rated as “high grade” on the malignancy scale; in 1 patient “low grade”, but the size of the tumor was beyond the possibility of resection. Postoperative histopathological examinations revealed that 17 patients had urothelial carcinoma of the bladder; 4 patients did not have cancer due to previous treatments, 3 patients were cured with neoadjuvant chemotherapy, and massive adenocarcinoma infiltration was identified in 1 patient. Neoadjuvant chemotherapy was used in 6 patients, and adjuvant chemotherapy in 7 patients. The patients received the same type of chemotherapy, cisplatin and gemcitabine, with modified doses depending on the results of morphology. Nine patients from the study group suffered from coexisting prostate cancer, and in 1 patient the cause of the problem was primary lung cancer. Parastomal hernias were observed in 2 patients in the postoperative course. From the surveyed group, after the research was completed, information about the deaths of 8 patients who took part in the study was obtained.

## 4 Results

### 4.1 Comparison of fatigue intensity in four measurements

**Fig. 1:**
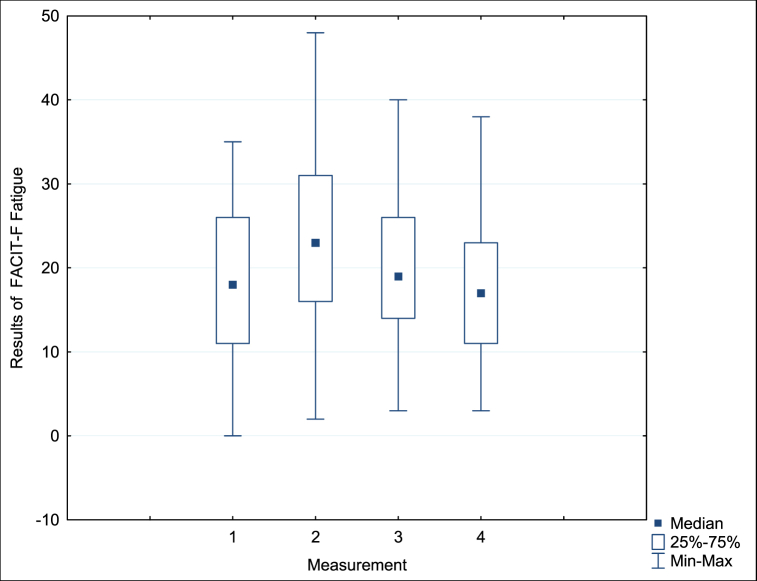
Fatigue intensity (FACIT-F) in the study group across four consecutive measurements with an interval of 3 months.

Statistically significant changes in the intensity of fatigue were observed during the study period, which allowed us to describe the dynamics of fatigue in the study group. The Kruskal-Wallis test identified differences in subjectively perceived fatigue in subsequent measurements (Chi^2 ANOVA (N = 21, df = 3) = 11.59 p = 0.008). The results of our analyses are presented in Fig. 1, from which we can conclude that patients reported varying degrees of fatigue during treatment.

Statistically significant changes in the intensity of fatigue were observed during the study period, which allowed us to describe the dynamics of fatigue in the study group. The Kruskal-Wallis test identified differences in subjectively perceived fatigue in subsequent measurements (Chi^2 ANOVA (N = 21, df = 3) = 11.59 p = 0.008). The results of our analyses are presented in Fig. 1, from which we can conclude that patients reported varying degrees of fatigue during treatment. Subsequent comparisons with the Wilcoxon sequence test revealed statistically significant differences between measurements 1 and 2 (p = 0.03), 2 and 4 (p = 0.004), and 3 and 4 (p = 0.03). The differences between measurements 1 and 3 (p = 0.28), 1 and 4 (p = 0.65), and 2 and 3 (p = 0.06) turned out to be statistically insignificant. Overall, six comparisons were made, and after adjusting the p-values using the Bonferroni method, the difference between measurements 3 and 4, 2 and 4 remained statistically significant. The remaining comparisons did not reach statistical significance after applying the Bonferroni correction at the significance level of 0.0083.

### 4.2 Correlations between fatigue and the subjective sense of the impact of disease on life in four measurements

Table 2 presents the correlations (Spearman's Rho) between subjectively perceived fatigue at subsequent stages of the study and health-related variables. Using a self-constructed question to measure the perception of the extent to which the disease affects the daily life of the respondents (answers from 0 “no effect at all” up to 10 “powerful impact”), we did not find any associations with fatigue at the first stage of the study. However, in subsequent measurements, a positive correlation emerged between the reported intensity of fatigue and the perceived impact of the disease on patients' lives (Rho = 0.71 for the second measurement; 0.45 for the third, and 0.51 for the fourth measurement, respectively, p < 0.05). When comparing these correlations across the three mentioned measurements, no statistically significant differences were observed—they remained at a similar level (p > 0.05).

**Table 2:** Correlations (Rho Spearman) between health and fatigue in four consecutive measurements.

	FACIT-F			
	Measurement 1	Measurement 2	Measurement 3	Measurement 4
The subjective impact of the disease on life	0.38	0.71^a^	0.45^a^	0.51^a^
NCCN-FACT FBISI-18 (general result)	0.61^a^	0.93^a^	0.83^a^	0.91^a^
DRS-Physical	0.56^a^	0.89^a^	0.70^a^	0.81^a^
Pain (GP4)	0.23	0.58^a^	0.63^a^	0.77^a^
Losing weight (C2)	0.43	0.67^a^	0.55^a^	0.35
Trouble with controlling urine (BL1)	−0.25	0.38	−0.04	0.51^a^
Feeling weak (H12)	0.22	0.92^a^	0.56^a^	0.56^a^
Dizziness (An9)	0.54^a^	0.64^a^	0.66^a^	0.57^a^
Trouble meeting the needs of the family (GP3)	0.76^a^	0.41	0.69^a^	0.59^a^
Appetite (C6)	0.46^a^	0.57^a^	0.44^a^	0.62^a^
Sleeping (Gf5)	0.38	0.72^a^	0.42	0.47^a^
TSE (Treatment Side Effects)	0.64^a^	0.94^a^	0.87^a^	0.87^a^
Nausea (GP2)	0.22	0.33	0.48*	0.63*
Lack of energy (GP1)	0.38	0.91^a^	0.78^a^	0.69^a^
Feeling ill (GP6)	0.71^a^	0.84^a^	0.78^a^	0.73^a^
Control of bowels (C3)	0.39	0.42	0.53^a^	0.64^a^
Bothered by side effects of treatment (GP5)	0.45^a^	0.69^a^	0.62^a^	0.52^a^

a p < 0.05.

### 4.3 Correlations between fatigue and the overall NCCN-FACT FBISI-18 (symptoms, condition, and well-being)

The results obtained using the NCCN-FACT FBISI-18 questionnaire, which concerns symptoms associated with cancer, are also summarized in the same Table 2. The study revealed statistically significant correlations between fatigue and experiencing symptoms of cancer at each stage of the study, with the strongest correlations in the second and fourth measurements (Rho = 0.93; 0.91, p < 0.05) compared to the correlation from the first measurement. In correlation comparisons, statistically significant differences between correlations were obtained between correlations from measurements 1 and 2 p = 0.007 and 1 and 4 p = 0.018).

### 4.4 Correlations between fatigue and disease-related symptoms

Further analyses were conducted using subscales of the NCCN-FACT FBISI-18 to identify cancer-related symptoms particularly associated with fatigue. The first subscale, Disease Related Symptoms - Physical (DRS-P), statistically significantly correlated with fatigue at each stage of the study. As symptoms related to physical functioning during cancer treatment increased in severity, so did fatigue (Rho = 0.56; 0.89; 0.83; 0.81, p < 0.05). At each stage of the measurement, the experience of dizziness (Rho = 0.54; 0.64; 0.66; 0.57, p < 0.05) and lack of appetite (Rho = 0.46; 0.57; 0.44; 0.62, p < 0.05) were statistically significantly correlated with fatigue. In the second, third, and fourth measurements, other symptoms correlated with fatigue, such as experiencing pain (Rho = 0.58; 0.63; 0.77, p < 0.05) and feeling weak (Rho = 0.92; 0.56; 0.56, p < 0.05). Experiencing weight loss correlated with fatigue in a statistically significant way in the second and third measurements (Rho = 0.67; 0.55, p < 0.05), and the sense of difficulty in meeting the needs of the family due to the patient's physical condition correlated with the intensity of fatigue in the first, third, and fourth measurements (Rho = 0.76; 0.69; 0.59, p < 0.05), and experiencing sleepiness in the second and fourth measurements (Rho = 0.72; 0.47, p < 0.05).

The highest number of correlations with fatigue occurred during the fourth measurement. Fatigue was statistically significantly correlated with all possible physical symptoms of cancer, apart from weight loss.

The strongest correlations (judging by the size of the correlation coefficient) between fatigue and experienced symptoms included the correlations between fatigue and a sense of weakness (Rho = 0.92, p < 0.05) and sleepiness (Rho = 0.72, p < 0.05) during the second measurement, pain in the third measurement (Rho = 0.77, p < 0.05) and the feeling of experiencing difficulties in meeting the needs of the family due to the physical condition in the first measurement (Rho = 0.76, p < 0.05).

### 4.5 Correlations between fatigue and treatment side effects

The scores from the Treatment Side Effects (TSE) subscale were also found to be correlated with fatigue, with an observable statistically significant difference between the two correlation coefficients between the first and second measurements (p = 0.005). Throughout all four stages of treatment, fatigue was correlated with the feeling of being sick (Rho = 0.71; 0.84; 0.78; 0.73, p < 0.05) and the annoyance experienced due to treatment side effects (Rho = 0.45; 0.69; 0.62; 0.52, p < 0.05).

There is an evident increase in the number of fatigue correlates among the side effects experienced in each subsequent measurement. In the third and fourth measurements, greater experiences of nausea, lack of energy, feeling sick, lack of control over bowel movements, and persistent side effects of treatment were associated with higher levels of fatigue. Refer to Table 2.

### 4.6 Correlations between fatigue and psychological variables

Table 3 presents the correlations between the experienced fatigue and variables related to the mental state: stress, anxiety, depressive symptoms and irritability. During the first measurement, no statistically significant correlations were observed between any of these variables and fatigue. However, during the second measurement, we observed a correlation between fatigue and stress (Rho = 0.76, p < 0.05), as well as a statistically significant correlation with anxiety (Rho = 0.57, p < 0.05) and symptoms of depression (Rho = 0.56, p < 0.05). In the third and fourth measurements, all these variables were statistically significantly correlated with fatigue, with the addition of irritability. There were no statistically significant differences between the correlation coefficients in the second, third and fourth measurements.

**Table 3:** Correlations between variables related to mental state and fatigue in four consecutive measurements.

	FACIT-F			
	Measurement 1	Measurement 2	Measurement 3	Measurement 4
PSS-10 (stress)	0.36	0.76^a^	0.65^a^	0.69^a^
HADS-A (anxiety)	0.39	0.57^a^	0.61^a^	0.73^a^
HADS-D (depression)	0.42	0.56^a^	0.61^a^	0.81^a^
HADS-R (irritability)	0.23	0.005	0.54^a^	0.45^a^

a p < 0.05.

### 4.7 The difference in fatigue between the surviving and non-surviving groups

In the final stage of analysis, the intensity of fatigue from subsequent measurements was compared between the group that died during the study (N = 8) and the group of people who survived over 6 months after the end of the study (N = 13). Using the Mann-Whitney *U* test, statistically significant differences were observed in the first, second, and third measurements regarding the intensity of fatigue between the two groups. Patients from the surviving group exhibited significantly lower fatigue levels in each of these measurements. The results are presented in Table 4.

**Table 4:** Comparison of the group that survived and died during the study in terms of the intensity of fatigue experienced using the Mann-Whitney *U* test.

		Group that survived (N = 13) Median	Group that died (N = 8) Median	U	Z	p
FACIT-F	Measurement 1	15.0	25.5	14.50	2.10	0.03^a^
	Measurement 2	23.0	32.0	16.0	1.97	0.04^a^
	Measurement 3	17.0	26.0	16.5	1.92	0.04^a^
	Measurement 4	16.0	22.0	22.5	1.40	0.16

Z – standardised value, U – rank sum.

a p < 0.05.

### 4.8 Comparison of fatigue in the group receiving and not receiving chemotherapy

The results obtained and presented above appear particularly significant in the context of the absence of statistically significant differences in terms of fatigue between the group receiving chemotherapy (neoadjuvant and adjuvant) and the group not receiving chemotherapy at each stage of the conducted measurements. These data are presented in Table 5 below.

**Table 5:** Comparison of the chemotherapy-treated group (neoadjuvant and adjuvant) and the non-chemotherapy group in terms of fatigue at all measurement stages using the Mann-Whitney *U* test.

		Group receiving neoadjuvant chemotherapy (N = 6) Median	Group not receiving neoadjuvant chemotherapy (N = 15) Median	U	Z	p
FACIT-F	Measurement 1	17.5	18.0	39.00	0.43	0.67
	Measurement 2	15.5	25.0	30.50	1.09	0.27
	Measurement 3	16.5	23.0	25.5	1.48	0.14
	Measurement 4	18.0	18.0	42.5	0.16	0.16
		Group receiving adjuvant chemotherapy (N = 7) Median	Group not receiving adjuvant chemotherapy (N = 14) Median	U	Z	p
FACIT-F	Measurement 1	18.0	17.5	51.00	0.18	0.86
	Measurement 2	25.0	22.5	52.50	0.07	0.94
	Measurement 3	21.0	18.0	51.5	0.14	0.89
	Measurement 4	19.0	16.5	51.5	0.14	0.89

*p < 0.05 Z – standardised value, U – rank sum.

## 5 Discussion

In this study, employing multiple measurements and post-project data, we were able to outline the dynamics of fatigue in a cohort of patients and relate our findings to patient survival. The patients under investigation constituted a relatively homogenous group facing similar circumstances (post-diagnosis and prior treatment, undergoing cystectomy, a radical procedure for bladder cancer).

This study revealed statistically significant fluctuations in fatigue assessments over approximately one year following cystectomy. This indicates the necessity for ongoing evaluation of patient's fatigue, without presuming that their condition will remain static throughout the treatment continuum. These findings align with contemporary recommendations for constant monitoring and patient education regarding fatigue in oncology [22,23].

Our study also aimed to identify factors associated with fatigue in oncology patients. We found significant correlations between fatigue and disease symptoms experienced by the patients at each stage of the measurements, with the strongest correlations during the second and fourth measurements. Physical complaints, notably dizziness and loss of appetite, were consistently linked with fatigue at each stage of treatment; while pain and weakness emerged as additional contributors during the second, third, and fourth stages. The fourth assessment exhibited the highest number of fatigue correlations.

Moreover, reported treatment side effects demonstrated a consistent association with fatigue across all four stages, particularly in the form of feeling illness and weariness associated with experiencing treatment-related side effects. With subsequent measurements, a growing number of correlates of fatigue were observed in the subscale of TSE (treatment side effects). A published study involving 99 bladder cancer patients after radical cystectomy described a similar interrelated cluster of symptoms. One of these symptoms pertained to fatigue or malaise and included feelings of tiredness, drowsiness, pain, memory problems, and loss of appetite. The same study revealed significant associations between the three symptom clusters identified and patients' quality of life. The strongest correlations were observed between quality of life and psycho-urinary problems, which included, among others, feelings of sadness and distress [24].

We also explored the associations between fatigue and psychological/mental health variables. Across all measurements except the initial one, fatigue demonstrated associations with stress, anxiety, depression, and irritability, highlighting their increasing significance and progressively detrimental impact on the long-term treatment process. This trend was particularly notable in the case of depressive symptoms, where the correlation between fatigue and depression strengthened in subsequent measurements, increasing from Rho = 0.56 in the second to Rho = 0.81 in the final measurement. These findings are consistent with the responses we received in our study-designed survey. Interestingly, there was no correlation between answers to the question about how the disease affects life and fatigue in the first measurement. In contrast, in the second, third, and fourth measurements, we obtained statistically significant correlations. Similar results demonstrating a connection between depression and cancer-related fatigue were obtained by many different authors [[25], [26], [27], [28]]. Furthermore, some researchers propose considering depression and fatigue as another example of a symptom cluster. For instance, the authors of longitudinal studies of these phenomena in women treated for breast cancer [29]. This is particularly relevant given research indicating a high prevalence of depressive symptoms, especially in elderly patients with bladder cancer [30].

We also encountered numerous reports linking stress and anxiety to cancer-related fatigue. A meta-analysis even highlighted stress as the most significant variable contributing to fatigue, leading to the recommendation for regular assessment of the emotional distress of patients treated for cancer [31]. Additionally, data from another systematic review, suggest that anxiety, alongside depression, may play a key role in cancer-related fatigue [32].

All three factors mentioned - depression, anxiety, and stress – significantly affect the overall functioning of the human body and are closely associated with the cancer-related fatigue [33]. A recent systematic review emphasised the need for further research not only on physical but also on mental health aspects, given their significant impact following treatment for bladder cancer [34].

Our goal was not to identify the specific variables that are more critical for fatigue, but rather to underscore the significance of various physical and mental factors that may contribute to its development. Some scientific studies in this field propose the idea of a multidimensional model, which is crucial for understanding the presence of significant fatigue. Among these studies, one highlighted that both physical factors (such as feeling drowsy, dyspnea, pain, and lack of appetite) and psychological factors (such as feeling sad, and irritatated) individually entered into the analysis are very good predictors of cancer-related fatigue. All the models generated from these analyses were statistically significant and emphasised the importance of both physical and psychological factors in explaining a patient's fatigue [35].

## 6 Strengths and limitations of the study

One of the primary limitations of the study is the relatively small size of the study group, which precluded conducting a full analysis in subgroups. The study group was largely homogeneous, comprising individuals diagnosed with bladder cancer who underwent cystectomy). Achieving absolute homogeneity in clinical settings is challenging; hence, we included patients with concurrent diagnoses of bladder and prostate cancer in our study, due to the frequent co-occurrence of these cancers and similar treatment protocols. Additionally, one patient with concomitant lung cancer was also included. It is essential to consider the clinical characteristics of the group when interpreting the findings. Further heterogeneity was introduced by variations in treatment modalities; some patients did not receive chemotherapy, while others were scheduled for such treatment. Nevertheless, our analyses revealed no association between chemotherapy and fatigue at any of the measurement stage, allowing us to explore other variables in our analyses.

We also wish to highlight the tremendous commitment of the patients. Participation in the longitudinal study, with multiple measurements, required their time and effort, which they were willing to invest, recognizing the importance of the fatigue topic. Our study is a pilot that we would like to continue further. We hope that our findings will serve as a catalyst for additional research by other investigators.

## 7 Conclusions

Systematic evaluation of fatigue is essential, and tailored treatments should be implemented based on the specific underlying causes identified in each patient [36]. This underscores the importance of an interdisciplinary approach involving a healthcare team working with oncological patients to address cancer-related fatigue [37]. It is especially important in the context of our result describing the relationship between fatigue and mortality. The group that survived at least 6 months after the end of the study were those who reported lower levels of fatigue in three out of four measurements. While, there is a pressing need for more research on the relationships between cancer-related fatigue and mortality, a similar result has been reported by other authors, indicating a correlation between survival time and cancer-related fatigue in a group of patients with advanced colorectal cancer and endometrial cancer [38]. Investigating fatigue and its associated variables, including potential causes, can raise awareness about the importance of addressing fatigue in the treatment process and help in the development of support programs for patients.

## Ethics and consent statement

This study was reviewed and approved by Bioethics Committee for Scientific Research in Medical University of Gdansk (Poland) with the approval number: Resolution No. NKBBN/452/2022. All participants provided written informed consent for the publication of their anonymised case details.

## Data availability statement

Data associated with the study have not been deposited into a publicly available repository because the current article is part of a broader scientific project. Upon the publication of all articles from this project, the data will be made available.

## CRediT authorship contribution statement

**Agata Zdun – Ryżewska:** Writing – review & editing, Writing – original draft, Validation, Supervision, Methodology, Formal analysis, Conceptualization. **Teresa Gawlik-Jakubczak:** Writing – review & editing, Project administration, Methodology, Data curation, Conceptualization. **Agnieszka Trawicka:** Writing – review & editing, Project administration, Investigation, Data curation, Conceptualization. **Paweł Trawicki:** Writing – review & editing, Software, Project administration.

## Declaration of competing interest

The authors declare that they have no known competing financial interests or personal relationships that could have appeared to influence the work reported in this paper.
